# Crystal structure and Hirshfeld surface analysis of (*E*)-2-[2-(2-amino-1-cyano-2-oxo­ethyl­idene)hydrazin-1-yl]benzoic acid *N*,*N*-di­methylformamide monosolvate

**DOI:** 10.1107/S2056989023011118

**Published:** 2024-01-05

**Authors:** Sevinc R. Hajiyeva, Fatali E. Huseynov, Zeliha Atioğlu, Mehmet Akkurt, Ajaya Bhattarai

**Affiliations:** a Department of Ecology and Soil Sciences, Baku State University, Z. Xalilov Str. 33, Az 1148 Baku, Azerbaijan; b Department of Aircraft Electrics and Electronics, School of Applied Sciences, Cappadocia University, Mustafapaşa, 50420 Ürgüp, Nevşehir, Türkiye; c Department of Physics, Faculty of Sciences, Erciyes University, 38039 Kayseri, Türkiye; d Department of Chemistry, M.M.A.M.C (Tribhuvan University) Biratnagar, Nepal; Illinois State University, USA

**Keywords:** crystal structure, hydrogen bonds, ring motifs, π–π stacking inter­actions, Hirshfeld surface analysis

## Abstract

In the title compound, C_10_H_8_N_4_O_3_·C_3_H_7_NO, the asymmetric unit contains two crystallographically independent mol­ecules *A* and *B*, each of which has one DMF solvate mol­ecule. Mol­ecules *A* and *B* both feature intra­molecular N—H⋯O hydrogen bonds, forming *S*(6) ring motifs and consolidating the mol­ecular configuration. In the crystal, N—H⋯O and O—H⋯O hydrogen bonds connect mol­ecules *A* and *B*, forming *R* ^2^_2_(8) ring motifs. Weak C—H⋯O inter­actions link the mol­ecules, forming layers parallel to the (  12) plane. The DMF solvent mol­ecules are also connected to the main mol­ecules (*A* and *B*) by N—H⋯O hydrogen bonds. π–π stacking inter­actions [centroid-to-centroid distance = 3.8702 (17) Å] between the layers also increase the stability of the mol­ecular structure in the third dimension. According to the Hirshfeld surface study, O⋯H/H⋯O inter­actions are the most significant contributors to the crystal packing (27.5% for mol­ecule *A* and 25.1% for mol­ecule *B*).

## Abstract 2

In the crystal, N—-H⋯O and O—H⋯O hydrogen bonds connect the two independent mol­ecules, forming 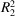 (8) ring motifs. Weak C—H⋯O inter­actions link the mol­ecules, forming layers parallel to the ( 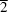 12) plane. The DMF solvent mol­ecules are also connected to the main mol­ecules N—H⋯O hydrogen bonds. π–π stacking inter­actions between the layers are also observed.

## 1. Chemical context

Aryl­hydrazones have been used extensively as substrates or ligands in the synthesis of organic or coordination compounds (Gurbanov *et al.*, 2022*a* ▸,*b* ▸; Khalilov *et al.*, 2021*a* ▸,*b* ▸; Kopylovich *et al.*, 2011 ▸). Depending on the position and nature of the substituent at the Ar–NH–N=synthon, and on the metal ion, different types of coordination compounds can be isolated, which have applications in catalysis, mol­ecular recognition, crystal growth and design, *etc*. (Afkhami *et al.*, 2019 ▸; Ma *et al.*, 2017 ▸, 2021 ▸; Mahmoudi *et al.*, 2017 ▸, 2021 ▸; Mahmudov *et al.*, 2010 ▸, 2023 ▸). Not only the hydrogen-bond donor or acceptor ability of the hydrazone moiety, but also the participation of the attached functional groups in various sorts of inter­molecular inter­actions improve their biological activities, catalytic performances, and reactivities (Martins *et al.*, 2017 ▸; Gurbanov *et al.*, 2017 ▸, 2020 ▸; Velásquez *et al.*, 2019 ▸). We have found that an aryl­hydrazone ligand can be produced by the activation of one cyano group on an active methyl­ene fragment of the parent mol­ecule to produce the carb­oxy amide moiety of the title mol­ecule, (*E*)-2-[2-(2-amino-1-cyano-2-oxo­ethyl­idene)hydrazin-1-yl]benzoic acid *N*,*N*-di­methyl­formamide monosolvate, which participates in inter­molecular hydrogen bonding in its crystal structure. 
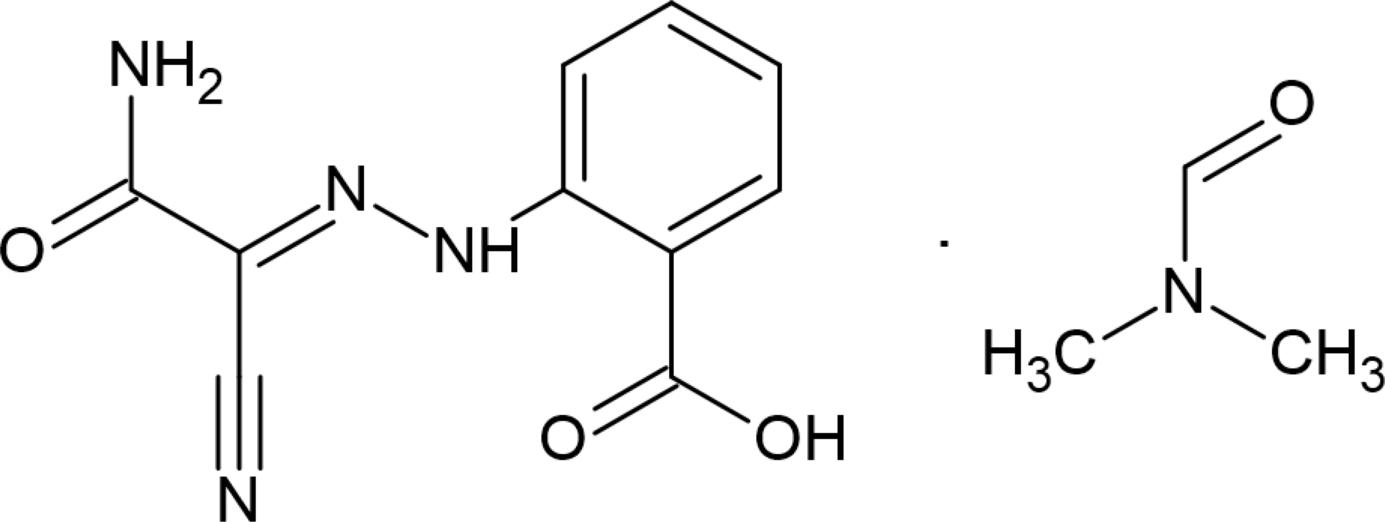


## 2. Structural commentary

**Figure 1:**
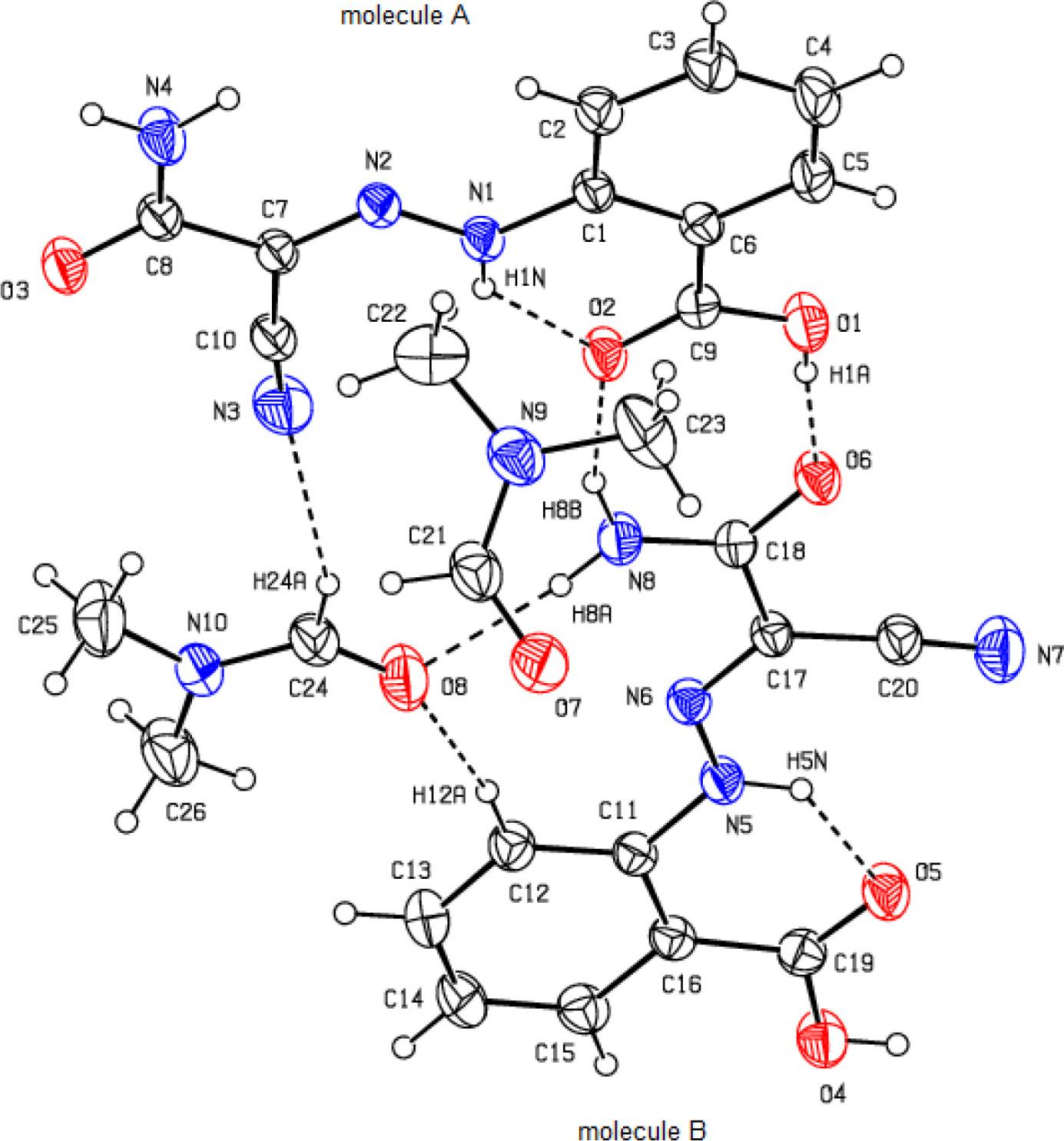
The asymmetric unit of the title compound. Displacement ellipsoids are drawn at the 30% probability level. The O—H⋯O, N—H⋯O and C—H⋯N hydrogen bonds are shown as dashed lines.

**Figure 2:**
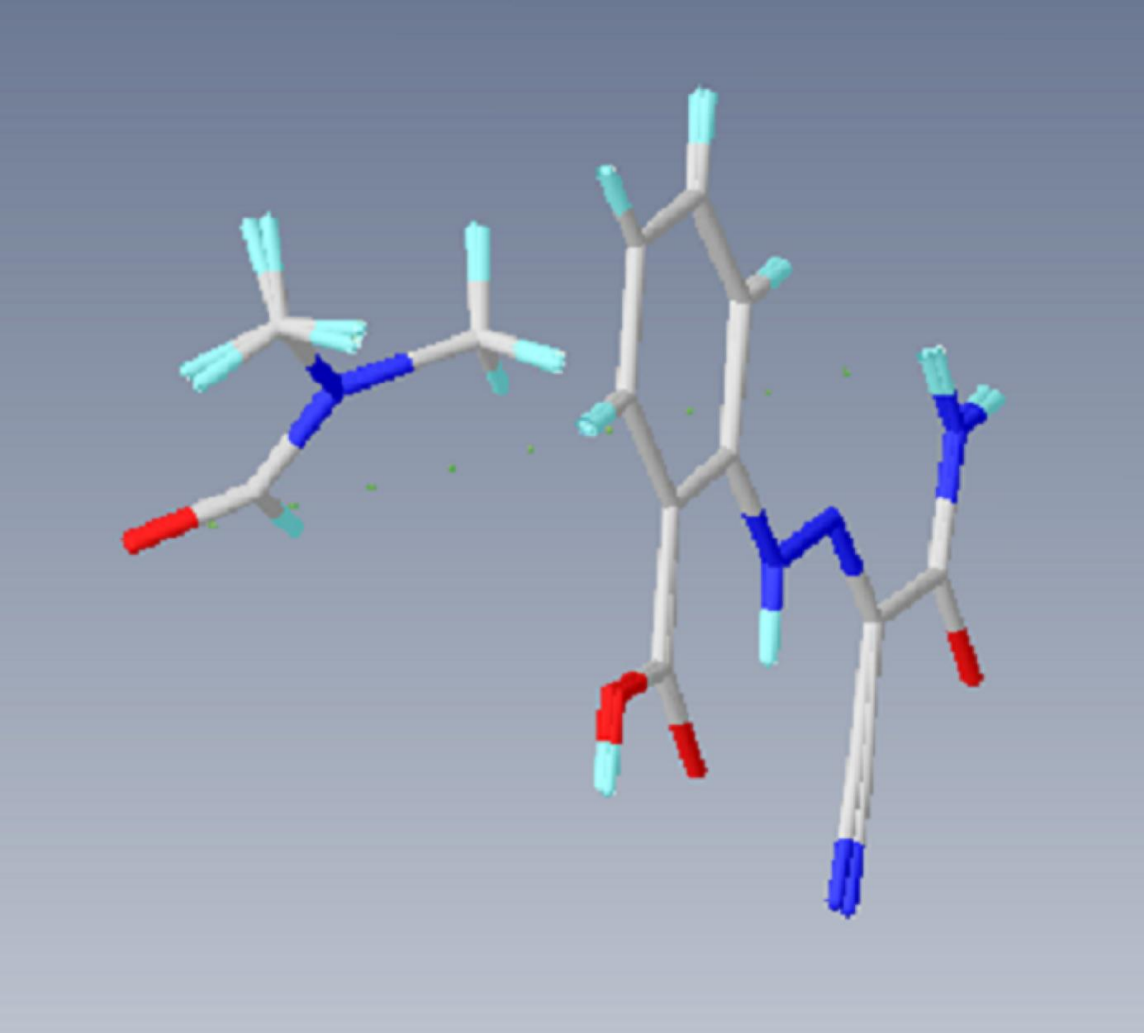
Image of the two independent mol­ecules *A* and *B* and the two solvent mol­ecules overlapping themselves in the asymmetric unit of the title compound. Color code: carbon (gray), hydrogen (white), nitro­gen (blue) and oxygen (red).

The asymmetric unit of the title compound, Fig. 1 ▸, contains two crystallographically independent main residue mol­ecules, *A* and *B*, and two di­methyl­formamide (DMF) solvate mol­ecules. As shown in Fig. 2 ▸ (r.m.s. deviation = 0.108 Å), mol­ecules *A* and *B* and their DMF solvent mol­ecules overlap quite well. The overlay diagram suggests possibly slight differences and *PLATON* ADDSYM (Spek, 2020 ▸) did not find any indication of pseudosymmetry with a cell of that volume. All attempts with CELL_NOW to find a twinned cell with the volume failed. In both mol­ecules *A* and *B*, intra­molecular N—H⋯O hydrogen bonds (Table 1 ▸) form *S*(6) ring motifs (Bernstein *et al.*, 1995 ▸), consolidating the mol­ecular configuration. The geometric properties of the title compound are normal and consistent with those of the related compounds listed in the *Database survey* (Section 4).

## 3. Supra­molecular features and Hirshfeld surface analysis

**Figure 3:**
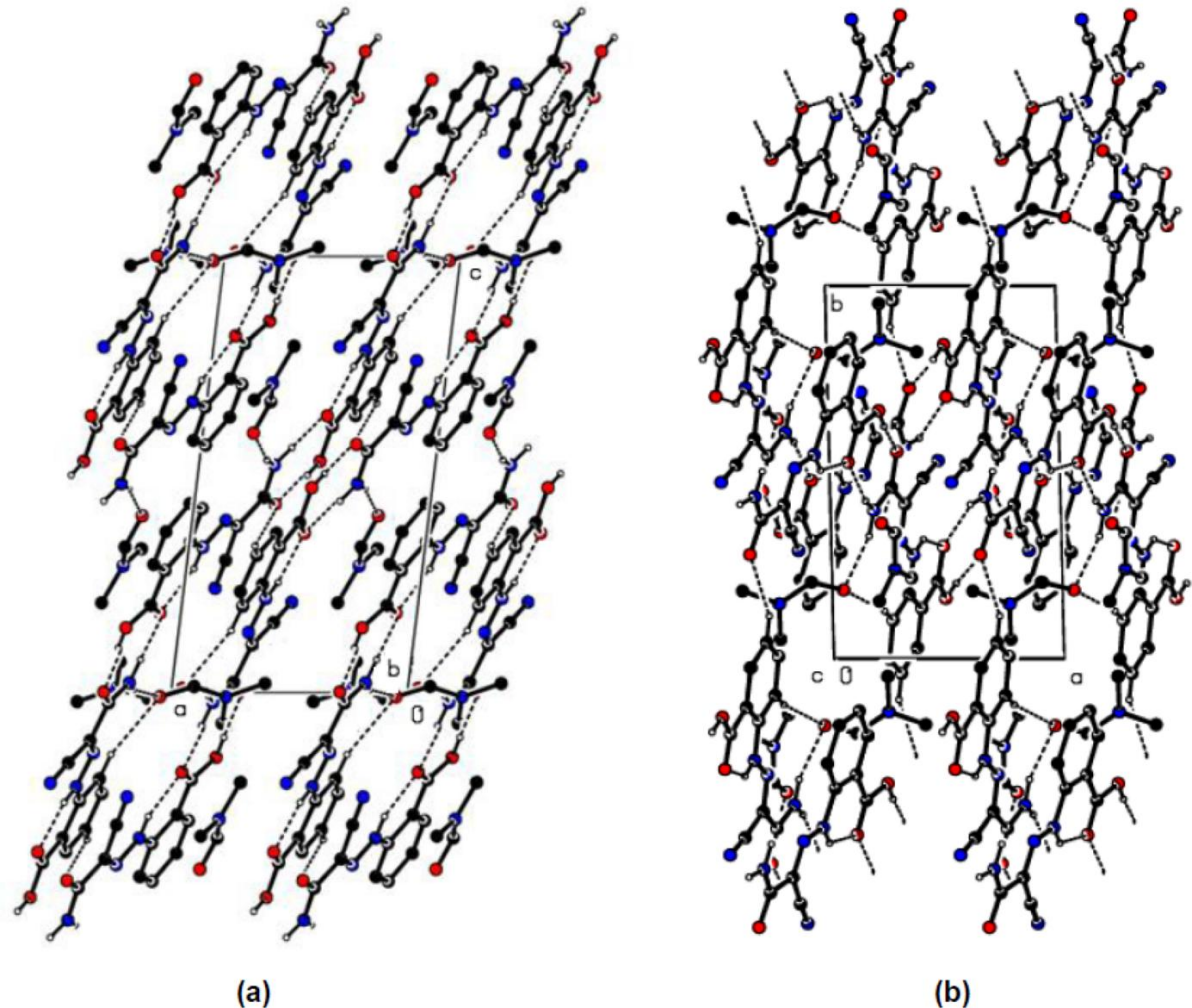
Partial packing diagrams, viewed down (*a*) the *b*-axis and (*b*) the *c*-axis. O—H⋯O, N—H⋯O, C—H⋯O and C—H⋯N hydrogen bonds are shown as dashed lines. H atoms not involved in these inter­actions have been omitted for clarity.

**Figure 4:**
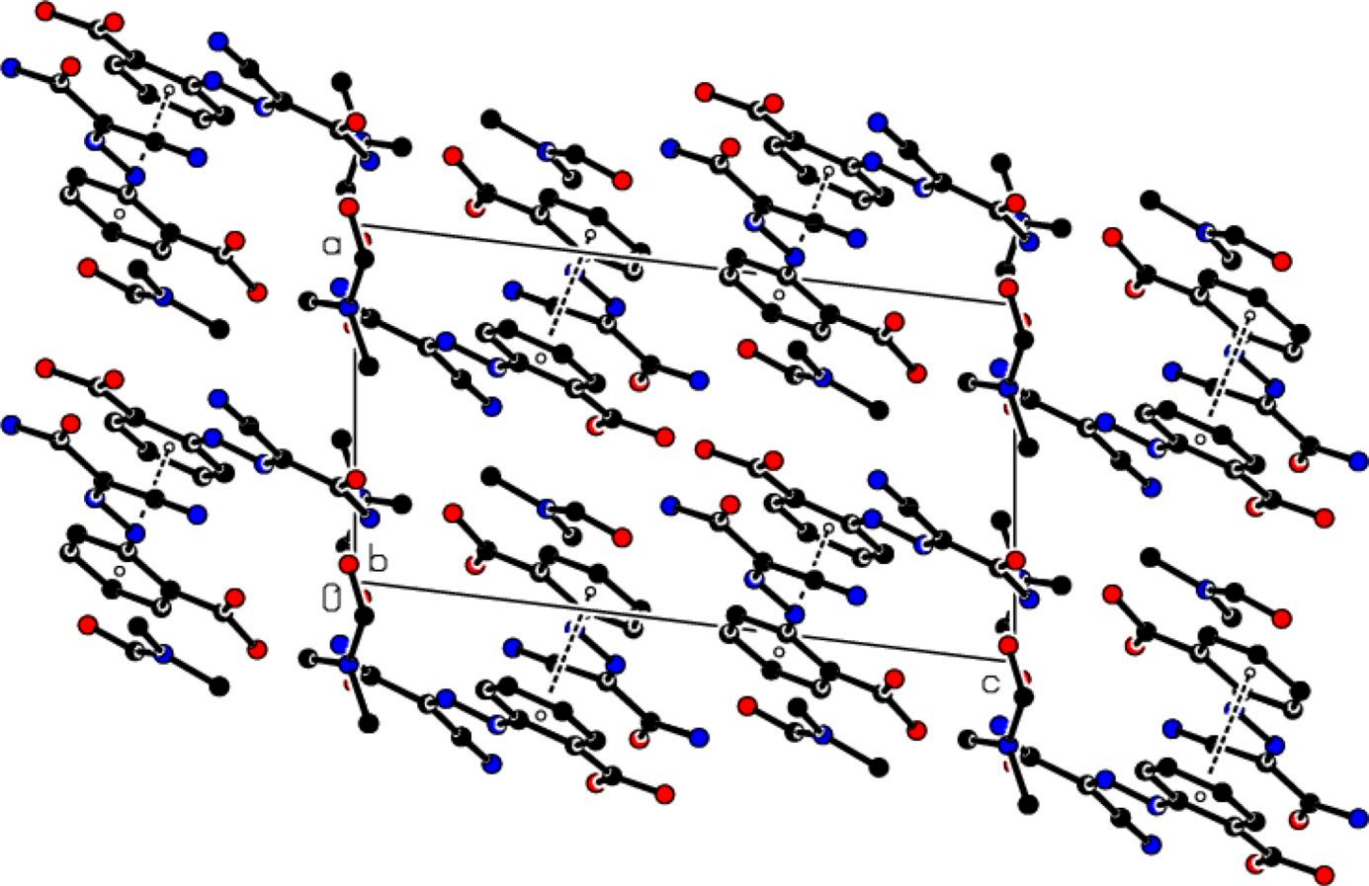
A partial packing diagram viewed down the *b*-axis. π–π stacking inter­actions are shown as dashed lines. H atoms have been omitted for clarity.

In the crystal, N—H⋯O and O—H⋯O hydrogen bonds (Table 1 ▸, Fig. 3 ▸ *a*,*b*) connect mol­ecules *A* and *B*, forming 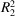 (8) ring motifs (Bernstein *et al.*, 1995 ▸). Weak C—H⋯O inter­actions link the mol­ecules, forming layers parallel to the ( 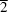 12) plane (Table 1 ▸, Fig. 3 ▸ *a,b*). The DMF solvent mol­ecules are also connected to the main mol­ecules (*A* and *B*) by N—H⋯O hydrogen bonds. π–π stacking inter­actions [*Cg*1⋯*Cg*2(1 − *x*, 1 − *y*, 1 − *z*) = 3.8702 (17) Å; *Cg*1 and *Cg*2 are the centroids of the (C1–C6) and (C11-C16) benzene rings of mol­ecules *A* and *B*, respectively] between the layers also increase the stability of the mol­ecular structure in the third dimension (Fig. 4 ▸).

**Figure 5:**
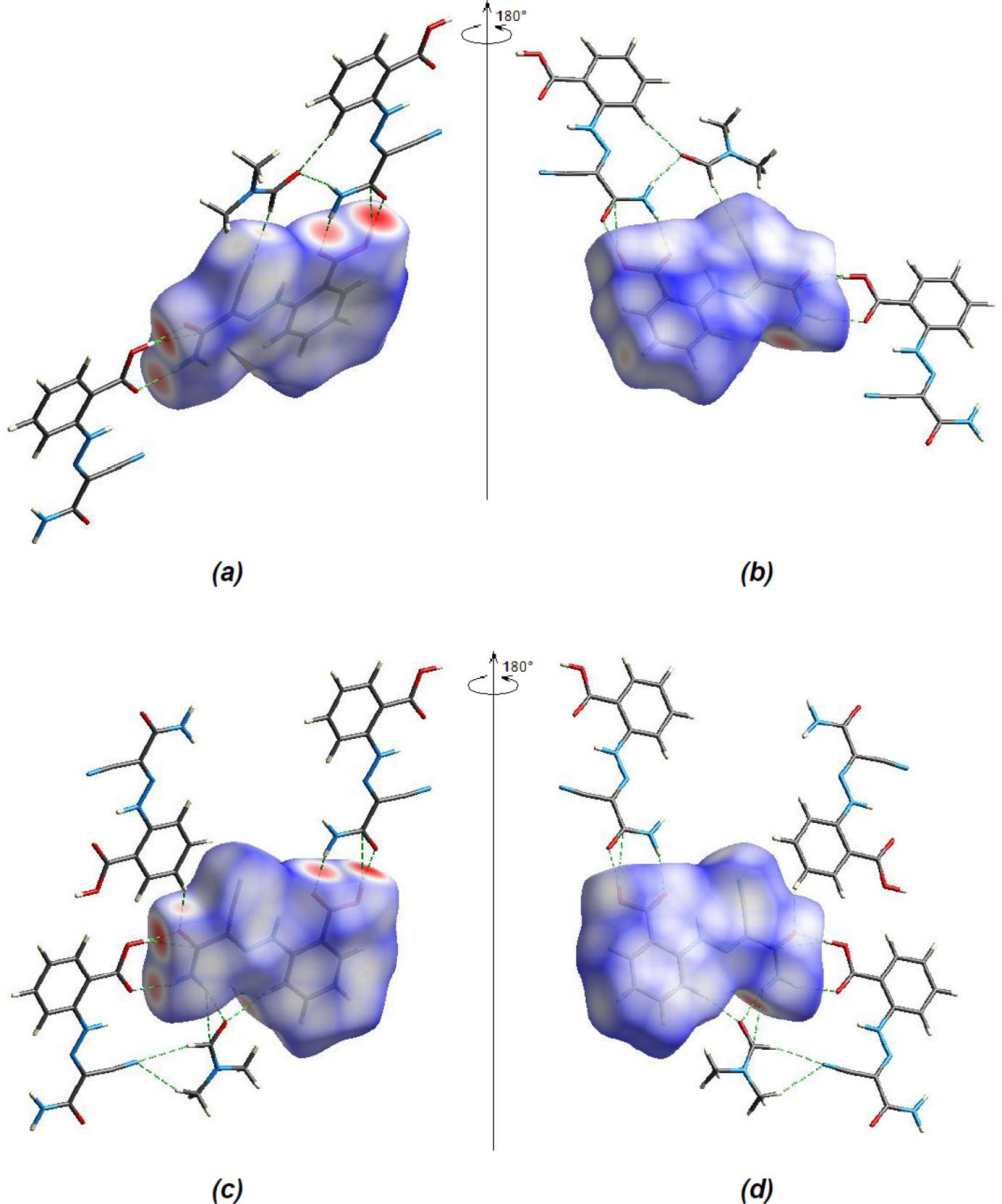
(*a*) Front and (*b*) back sides of the three-dimensional Hirshfeld surface of the title compound mapped over *d*_norm_ for *A*, (*c*) front and (*d*) back sides for *B*.

**Figure 6:**
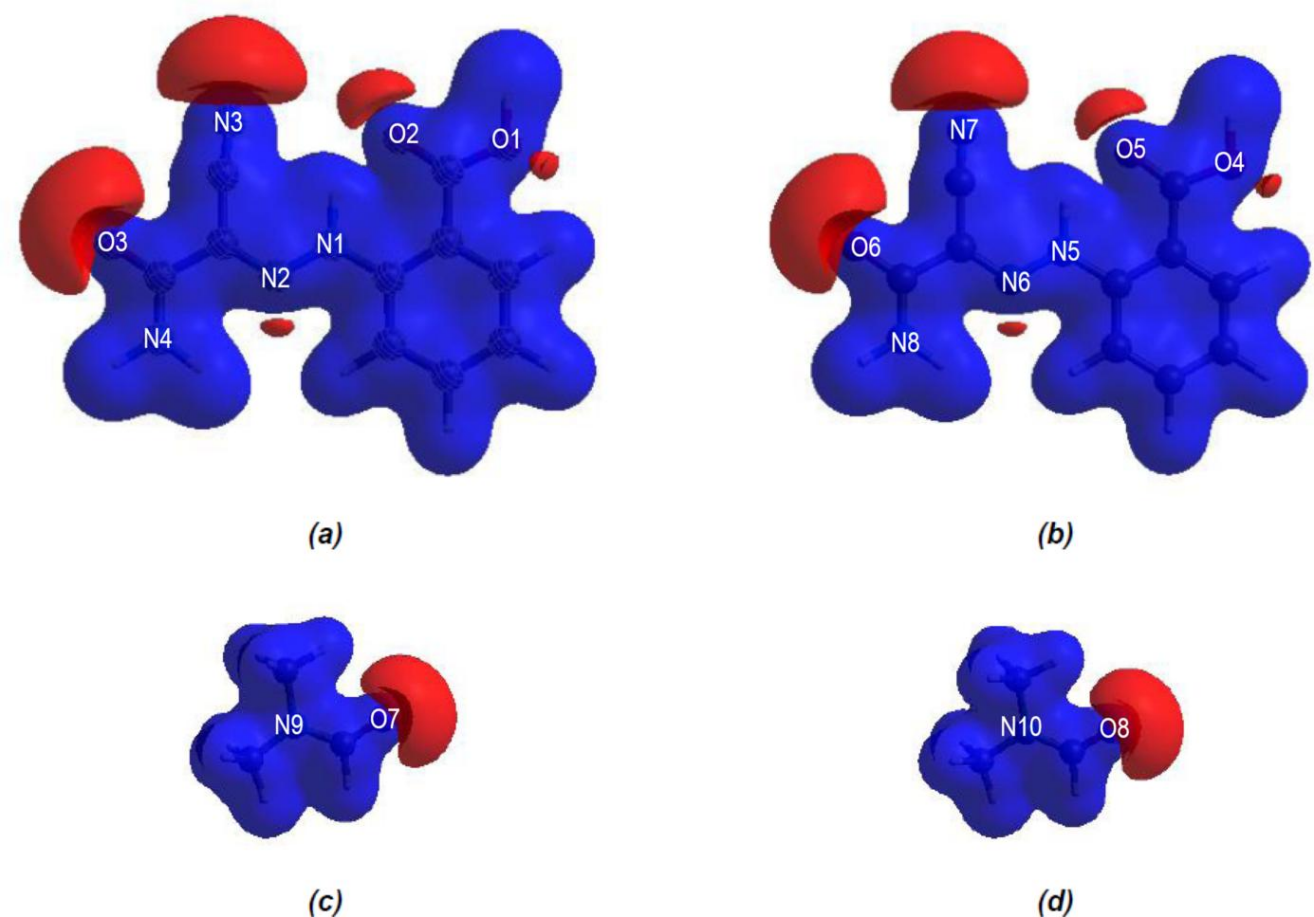
Views of the three-dimensional Hirshfeld surfaces of the four components in the asymmetric unit of the title compound plotted over electrostatic potential energy in the range −0.0500 to 0.0500 a.u., using the STO-3 G basis set at the Hartree–Fock level of theory: (*a*) mol­ecule *A*, (*b*) mol­ecule *B*, (*c*) DMF solvent mol­ecule (with O7), (*d*) DMF solvent mol­ecule (with O8).

**Figure 7:**
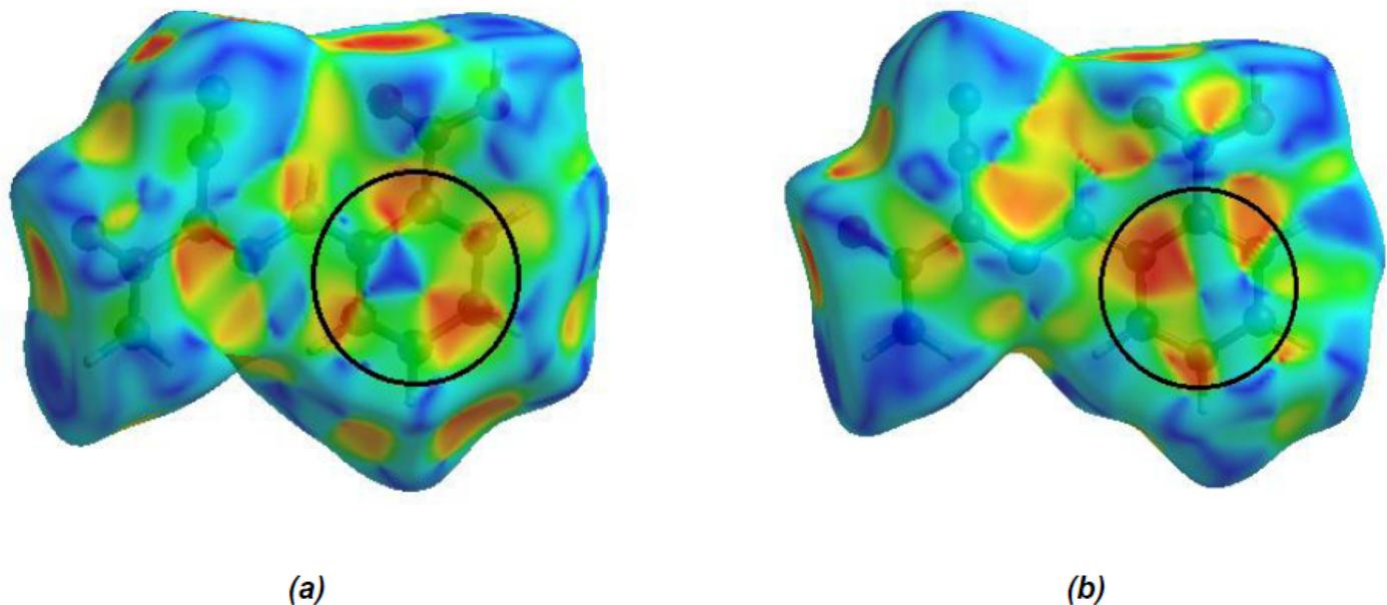
Hirshfeld surface of the title compound plotted over shape-index (*a*) for mol­ecule *A* and (*b*) for mol­ecule *B*.

In order to visualize the inter­molecular inter­actions in the crystal of the title compound, a Hirshfeld surface analysis was carried out using *CrystalExplorer 17.5* (Spackman *et al.*, 2021 ▸). In the Hirshfeld surface plotted over *d*_norm_ (Fig. 5 ▸), the white surface indicates contacts with distances equal to the sum of the van der Waals radii, and the red and blue colours indicate distances shorter (in close contact) or longer (distant contact) than the sum of the van der Waals radii (Venkatesan *et al.*, 2016 ▸). The bright-red spots indicate their roles as respective donors and/or acceptors; they also appear as blue and red regions corresponding to positive and negative potentials on the Hirshfeld surface mapped over electrostatic potential (Spackman *et al.*, 2008 ▸; Jayatilaka *et al.*, 2005 ▸), shown in Fig. 6 ▸. The blue regions indicate positive electrostatic potential (hydrogen-bond donors), while the red regions indicate negative electrostatic potential (hydrogen-bond acceptors). The shape-index of the Hirshfeld surface is a tool to visualize π–π stacking inter­actions by the presence of adjacent red and blue triangles (Fig. 7 ▸).

**Figure 8:**
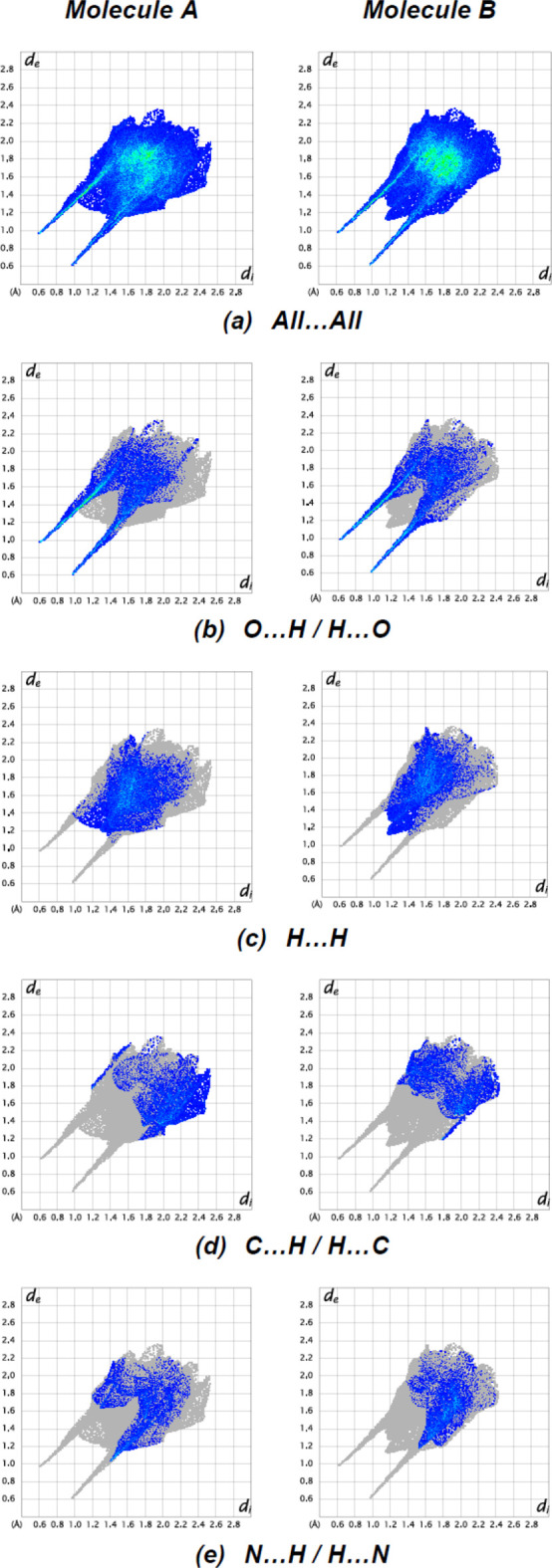
The full two-dimensional fingerprint plots for the title compound, showing (*a*) all inter­actions, and delineated into (*b*) O⋯H/H⋯H, (*c*) H⋯H, (*d*) C⋯H/H⋯C and (*e*) N⋯H/H⋯N inter­actions. The *d*_i_ and *d*_e_ values are the closest inter­nal and external distances (in Å) from given points on the Hirshfeld surface contacts.

The overall two-dimensional fingerprint plots for mol­ecules *A* and *B* are shown in (Fig. 8 ▸ *a*) and those delineated into O⋯H/H⋯O, H⋯H, C⋯H/H⋯C and N⋯H/H⋯N contacts (McKinnon *et al.*, 2007 ▸) are illustrated in Fig. 8 ▸ *b*–*e*, respectively, together with their relative contributions to the Hirshfeld surface. The most important inter­action is H⋯O/O⋯H (Table 2 ▸), contributing 27.5% (for *A*) and 25.1% (for *B*) to the overall crystal packing; this is shown in Fig. 8 ▸ *b* where the pairs of spikes have tips at *d*_e_ + *d*_i_ = 1.55 Å (for *A* and *B*). The H⋯H contacts contribute 27.3% for *A* and 24.3% for B to the Hirshfeld surface and are shown in Fig. 8 ▸ *c* as widely scattered points of high density due to the large hydrogen content of the mol­ecule with the tips at *d*_e_ = *d*_i_ = 2.50 Å (for *A*) and 2.25 Å (for *B*). The high contribution of these inter­actions suggest that van der Waals inter­actions play the major role in the crystal packing (Hathwar *et al.*, 2015 ▸). In the absence of C—H⋯π inter­actions, the pairs of distorted spikes in the fingerprint plots delineated into H⋯C/C⋯H contacts (Fig. 8 ▸ *d*; 15.4% for *A* and 15.3% for *B*) have the tips at *d*_e_ + *d*_i_ = 2.90 Å for *A* and 3.00 Å for *B*. The pair of distorted wings in the fingerprint plot delineated into N⋯H/H⋯N contacts (Fig. 8 ▸ *e*; 13.3% contributions for *A* and 14.0% for *B*) have the tips at *d*_e_ + *d*_i_ = 2.40 Å for *A* and 2.70 Å for *B*. The surroundings of mol­ecules *A* and *B* are very similar, as can be observed from a comparison of the supplied data.

**Figure 9:**
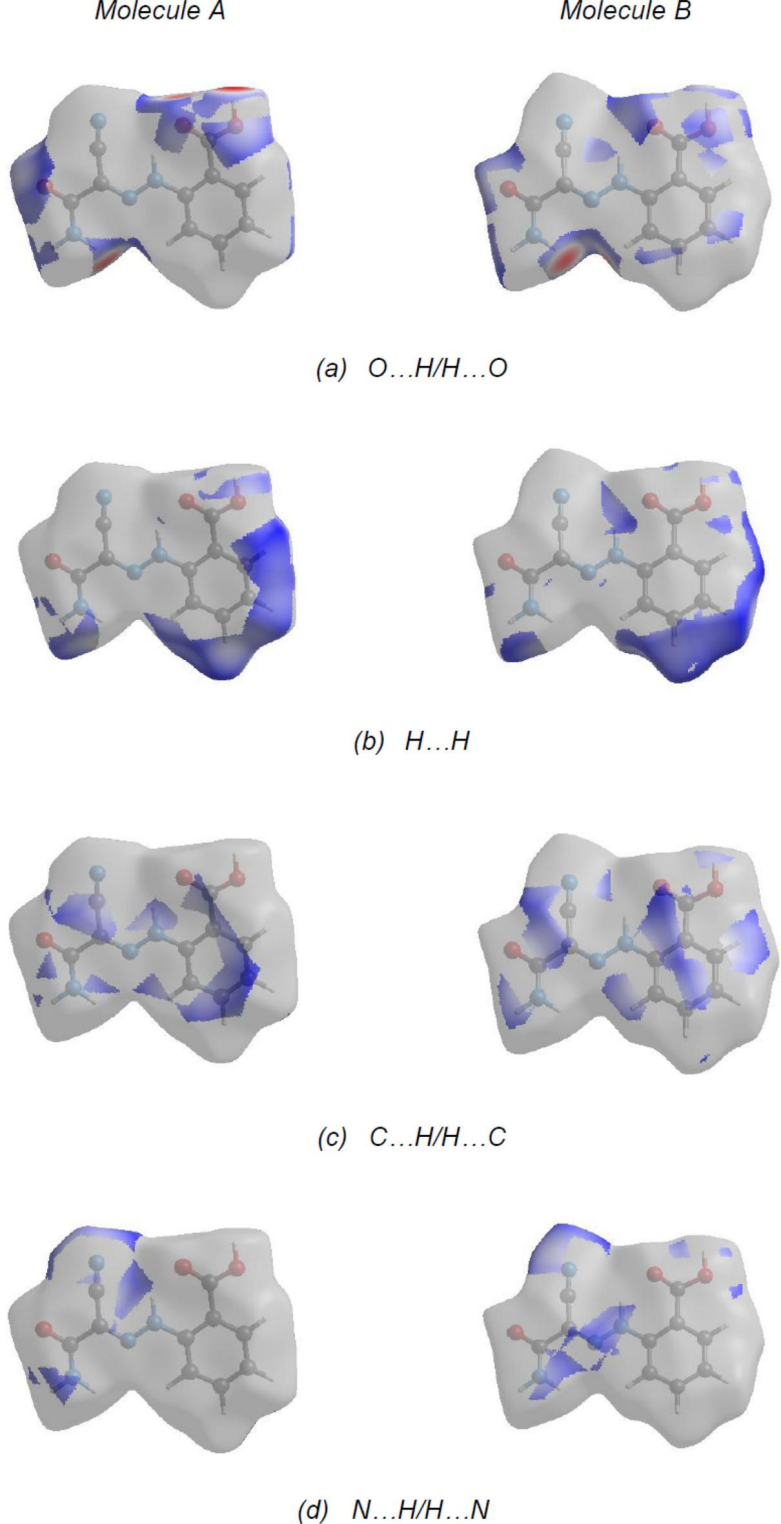
Hirshfeld surface representations with the function *d*_norm_ plotted onto the surface for (*a*) O⋯H/H⋯O, (*b*) H⋯H, (*c*) C⋯H/H⋯C and (*d*) N⋯H/H⋯N inter­actions.

The nearest neighbour coordination environment of a mol­ecule can be determined from the color patches on the Hirshfeld surface based on how close to other mol­ecules they are. The Hirshfeld surface representations with the function *d*_norm_ plotted onto the surface are shown for the O⋯H/H⋯O, H⋯H, C⋯H/H⋯C and N⋯H/H⋯N inter­actions in Fig. 9 ▸ *a*–*d*, respectively.

**Figure 10:**
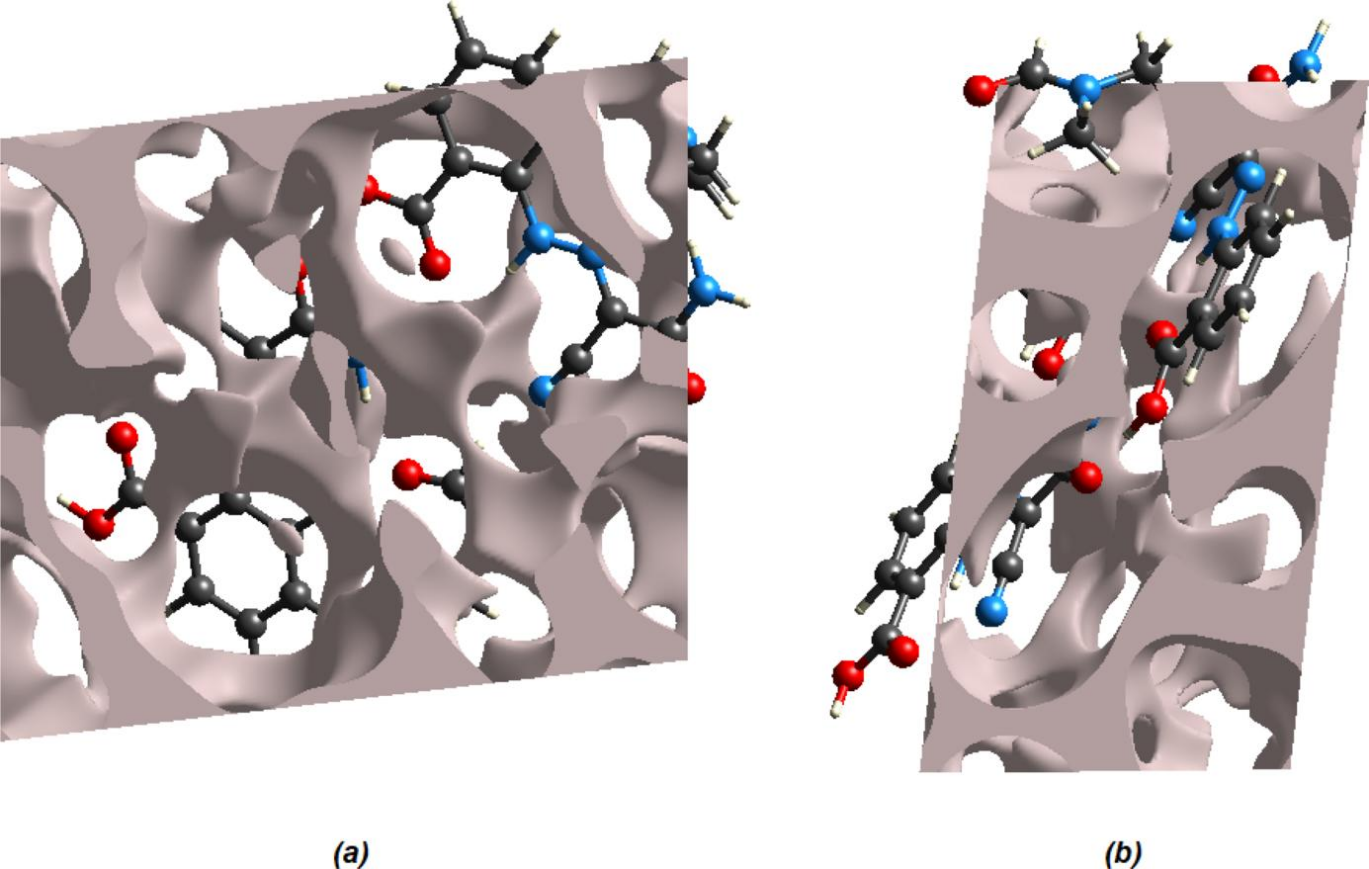
Graphical views of voids in the crystal packing of the title compound (*a*) along the *a*-axis direction and (*b*) along the *b*-axis direction.

The strength of the crystal packing is important for determining the response to an applied mechanical force. If the crystal packing results in significant voids, then the mol­ecules are not tightly packed and a small amount of applied external mechanical force may easily break the crystal. To check the mechanical stability of the crystal, a void analysis was performed by adding up the electron densities of the spherically symmetric atoms contained in the asymmetric unit (Turner *et al.*, 2011 ▸). The void surface is defined as an isosurface of the procrystal electron density and is calculated for the enclosed volume. The volume of the crystal voids (Fig. 10 ▸ *a*,*b*) and the percentage of free space in the unit cell are calculated as 178.70 Å^3^ and 11.93%, respectively. Thus, the crystal packing appears compact and the mechanical stability should be substantial.

## 4. Database survey

A search of the Cambridge Structural Database (CSD, Version 5.43, last update November 2022; Groom *et al.*, 2016 ▸) for the (2*E*)-2-cyano-2-hydrazinylideneacetamide unit yielded five compounds related to the title compound, *viz*. (*E*,*E*)-1-(2-hy­droxy­imino-1-phenyl­ethyl­idene)semicarbazide monohydrate (CSD refcode VORMEV; Öztürk *et al.*, 2009 ▸), 2-[(4,7-di­methyl­quinolin-2-yl)methyl­idene]hydrazine-1-carboxamide dihydrate (MIQPIO; Aydemir *et al.*, 2018 ▸), 2-(but-2-en-1-yl­idene)hydrazinecarboxamide (WOTRII; Arfan & Rukiah, 2015 ▸), 2-(pyridin-4-yl­methyl­ene)hydrazinecarboxamide hemihydrate (GUHXOY; Inoue *et al.*, 2015 ▸) and (*E*)-1-(4-meth­oxy­benzyl­idene)semicarbazide (YIFTOX; Liang *et al.*, 2007 ▸).

In the crystal of VORMEV, inter­molecular O—H⋯O and N—H⋯O hydrogen bonds link the mol­ecules, and 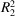 (8) ring motifs are apparent. In the crystal of MIQPIO, the mol­ecules are linked by O—H⋯O, N—H⋯O and O—H⋯N hydrogen bonds, forming a two-dimensional network parallel to (101). In the crystal of WOTRII, inter­molecular N—H⋯O hydrogen bonds link the mol­ecules into layers parallel to the *bc* plane. In the crystal of GUHXOY, mol­ecules are linked into an infinite three-dimensional network by classical N—H⋯O_s_ (s = semicarbazone) and O_w_—H⋯N (w = water) hydrogen bonds. In the crystal of YIFTOX, the almost planar mol­ecules inter­act by way of N—H⋯O hydrogen bonds.

## 5. Synthesis and crystallization

214 mg (1 mmol) of 2-[2-(di­cyano­methyl­ene)hydrazin­yl]benzoic acid was dissolved in 15 mL of acetone and 0.1 mL of water were added, then stirred at 353 K for 1 h. Then, the solvent was evacuated by a rotary evaporator, and the obtained yellow powder was crystallized in a mixture of acetone and di­methyl­formamide (20/1; *v*/*v*). Yield, 83%, yellow powder soluble in DMSO, methanol, ethanol and DMF. Analysis calculated for C_13_H_15_N_5_O_4_ (*M_r_ =* 305.29): C 51.15, H 4.95, N 22.94; found: C 51.13, H 4.91, N 22.92%. IR (KBr): 3211 *ν*(OH), 2948 and 2876 *ν*(NH), 2216 *ν*(CN), 1667 *ν*(C=O) and 1610 *ν*(C=N) cm^−1. 1^H NMR (300.130 MHz) in DMSO-*d*_6_, inter­nal TMS, *δ* (ppm): 2.66 and 2.83 (6H, 2CH_3_), 4.31 (1H, OH), 7.86 (2H, NH_2_), 8.18-8.56 (4H, Ar), 8.70 (1H, CH of DMF) and 14.46 (1H, N—H). ^13^C{^1^H} NMR (75.468 MHz, DMSO-*d*_6_). *δ*: 35.0 and 36.9 (2CH_3_), 109.8 (CN), 112.2 C—COOH, 115.5 (CH, Ar), 123.9 (CH, Ar), 128.5 (CH, Ar), 132.0 (CH, Ar), 133.4 (C=O), 143.5 (C—NH), 161.2 (COOH), 163.9 (C=O of DMF), 165.0 (C=O).

## 6. Refinement

Crystal data, data collection and structure refinement details are summarized in Table 3 ▸. C-bound H atoms were placed in geometrically calculated positions (C—H = 0.93 and 0.96 Å) and refined using a riding model with *U*_iso_(H) = 1.2*U*_eq_(C) for aromatic groups and *U*_iso_(H) = 1.5*U*_eq_(C) for methyl groups. N- and O-bound hydrogen atoms were located in difference-Fourier maps, but their positional parameters were fixed and their isotropic displacement parameters were refined with *U*_iso_(H) =1.2 or 1.5*U*_eq_(N,O). Two reflections, (010) and (202), affected by the beam stop, were omitted in the final cycles of refinement.

## Supplementary Material

Crystal structure: contains datablock(s) I. DOI: 10.1107/S2056989023011118/ej2001sup1.cif

Structure factors: contains datablock(s) I. DOI: 10.1107/S2056989023011118/ej2001Isup2.hkl

Supporting information file. DOI: 10.1107/S2056989023011118/ej2001Isup3.cml

CCDC reference: 2322557

Additional supporting information:  crystallographic information; 3D view; checkCIF report

## Figures and Tables

**Table 1:** Hydrogen-bond geometry (Å, °)

*D*—H⋯*A*	*D*—H	H⋯*A*	*D*⋯*A*	*D*—H⋯*A*
O1—H1*A*⋯O6	0.85	1.73	2.580 (3)	175
O4—H4*B*⋯O3^i^	0.85	1.75	2.590 (3)	171
N1—H1*N*⋯O2	0.90	1.89	2.617 (3)	136
N4—H4*C*⋯O7^ii^	0.90	2.04	2.915 (3)	162
N4—H4*D*⋯O5^iii^	0.90	2.09	2.952 (3)	161
N5—H5*N*⋯O5	0.90	1.93	2.640 (3)	134
N8—H8*A*⋯O8	0.90	2.01	2.889 (3)	164
N8—H8*B*⋯O2	0.90	2.14	2.990 (3)	158
C2—H2*A*⋯O7^ii^	0.93	2.60	3.505 (4)	165
C4—H4*A*⋯O6^iv^	0.93	2.52	3.372 (3)	153
C12—H12*A*⋯O8	0.93	2.39	3.311 (4)	171
C24—H24*A*⋯N3	0.93	2.62	3.498 (4)	158

Symmetry codes: (i) 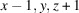 ; (ii) 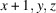 ; (iii) 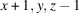 ; (iv) 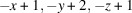 .

**Table 2:** Inter­atomic contacts (Å) for the title compound

Contact	Distance	Symmetry operation
H1*A*⋯O6	1.73	*x*, *y*, *z*
H5*A*⋯O1	2.74	1 − *x*, 2 − *y*, 1 − *z*
O3⋯H4*B*	1.75	1 + *x*, *y*, −1 + *z*
N3⋯C20	3.23	1 − *x*, 1 − *y*, 1 − *z*+
N3⋯H24*A*	2.62	*x*, *y*, *z*
H4*C*⋯O7	2.04	1 + *x*, *y*, *z*
H4*A*⋯O6	2.52	1 − *x*, 2 − *y*, 1 − *z*
O4⋯C21	3.21	-*x*, 1 − *y*, 1 − *z*
C20⋯N3	3.23	1 − *x*, 1 − *y*, 1 − *z*
H8*A*⋯O8	2.01	*x*, *y*, *z*

**Table 3:** Experimental details

Crystal data	
Chemical formula	C_10_H_8_N_4_O_3_·C_3_H_7_NO
*M*_r_	305.30
Crystal system, space group	Triclinic, *P* 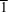
Temperature (K)	296
*a*, *b*, *c* (Å)	7.9481 (7), 12.8523 (13), 14.8737 (15)
α, β, γ (°)	96.651 (4), 96.960 (3), 90.548 (3)
*V* (Å^3^)	1497.6 (3)
*Z*	4
Radiation type	Mo *K*α
μ (mm^−1^)	0.10
Crystal size (mm)	0.35 × 0.23 × 0.15

Data collection	
Diffractometer	Bruker D8 Quest PHOTON 100 detector
Absorption correction	Multi-scan (*SADABS*; Krause *et al.*, 2015 ▸)
*T*_min_, *T*_max_	0.956, 0.975
No. of measured, independent and observed [*I* > 2σ(*I*)] reflections	51380, 6106, 4113
*R*_int_	0.083
(sin θ/λ)_max_ (Å^−1^)	0.627

Refinement	
*R*[*F* ^2^ > 2σ(*F* ^2^)], *wR*(*F* ^2^), *S*	0.063, 0.186, 1.12
No. of reflections	6106
No. of parameters	401
H-atom treatment	H-atom parameters constrained
Δρ_max_, Δρ_min_ (e Å^−3^)	0.28, −0.24

Computer programs: *APEX4* and *SAINT* (Bruker, 2018 ▸), *SHELXT* (Sheldrick, 2015*a* ▸), *SHELXL2018* (Sheldrick, 2015*b* ▸), *ORTEP-3 for Windows* (Farrugia, 2012 ▸) and *PLATON* (Spek, 2020 ▸).

## supplementary crystallographic information

### Crystal data

**Table:**

C_10_H_8_N_4_O_3_·C_3_H_7_NO	*Z* = 4
*M**_r_* = 305.30	*F*(000) = 640
Triclinic, *P*1	*D*_x_ = 1.354 Mg m^−^^3^
*a* = 7.9481 (7) Å	Mo *K*α radiation, λ = 0.71073 Å
*b* = 12.8523 (13) Å	Cell parameters from 9961 reflections
*c* = 14.8737 (15) Å	θ = 2.2–26.3°
α = 96.651 (4)°	µ = 0.10 mm^−^^1^
β = 96.960 (3)°	*T* = 296 K
γ = 90.548 (3)°	Plate, yellow
*V* = 1497.6 (3) Å^3^	0.35 × 0.23 × 0.15 mm

### Data collection

**Table:**

Bruker D8 Quest PHOTON 100 detector diffractometer	4113 reflections with *I* > 2σ(*I*)
φ and ω scans	*R*_int_ = 0.083
Absorption correction: multi-scan (SADABS; Krause *et al.*, 2015)	θ_max_ = 26.5°, θ_min_ = 2.6°
*T*_min_ = 0.956, *T*_max_ = 0.975	*h* = −9→9
51380 measured reflections	*k* = −16→16
6106 independent reflections	*l* = −18→18

### Refinement

**Table:**

Refinement on *F*^2^	Primary atom site location: structure-invariant direct methods
Least-squares matrix: full	Secondary atom site location: difference Fourier map
*R*[*F*^2^ > 2σ(*F*^2^)] = 0.063	Hydrogen site location: mixed
*wR*(*F*^2^) = 0.186	H-atom parameters constrained
*S* = 1.12	*w* = 1/[σ^2^(*F*_o_^2^) + (0.0525*P*)^2^ + 1.4706*P*] where *P* = (*F*_o_^2^ + 2*F*_c_^2^)/3
6106 reflections	(Δ/σ)_max_ < 0.001
401 parameters	Δρ_max_ = 0.28 e Å^−^^3^
0 restraints	Δρ_min_ = −0.24 e Å^−^^3^

### Special details

**Table:**

Geometry. All esds (except the esd in the dihedral angle between two l.s. planes) are estimated using the full covariance matrix. The cell esds are taken into account individually in the estimation of esds in distances, angles and torsion angles; correlations between esds in cell parameters are only used when they are defined by crystal symmetry. An approximate (isotropic) treatment of cell esds is used for estimating esds involving l.s. planes.

### Fractional atomic coordinates and isotropic or equivalent isotropic displacement parameters (Å^2^)

**Table:**

	*x*	*y*	*z*	*U*_iso_*/*U*_eq_	
O1	0.5077 (3)	0.83808 (16)	0.46961 (15)	0.0606 (6)	
H1A	0.461271	0.798067	0.501619	0.091*	
O2	0.5136 (3)	0.69907 (15)	0.36569 (14)	0.0526 (5)	
O3	0.7061 (3)	0.44678 (15)	−0.00144 (14)	0.0566 (6)	
O4	−0.2192 (3)	0.33799 (16)	0.85161 (15)	0.0617 (6)	
H4B	−0.240599	0.379454	0.897420	0.093*	
O5	−0.0804 (3)	0.47622 (16)	0.81769 (15)	0.0609 (6)	
O6	0.3548 (3)	0.72532 (15)	0.56897 (14)	0.0541 (5)	
O7	−0.0481 (4)	0.8179 (2)	0.0095 (2)	0.0812 (8)	
O8	0.2197 (4)	0.35888 (19)	0.40513 (18)	0.0809 (8)	
N1	0.6435 (3)	0.72330 (17)	0.21577 (15)	0.0425 (5)	
H1N	0.599503	0.681439	0.251926	0.051*	
N2	0.6978 (3)	0.68585 (17)	0.13792 (15)	0.0413 (5)	
N3	0.5278 (4)	0.4699 (2)	0.2074 (2)	0.0715 (8)	
N4	0.8120 (4)	0.6045 (2)	−0.02242 (18)	0.0626 (8)	
H4C	0.834937	0.673399	−0.006946	0.075*	
H4D	0.847177	0.579809	−0.075956	0.075*	
N5	0.0657 (3)	0.45672 (17)	0.66661 (16)	0.0452 (5)	
H5N	0.039820	0.498241	0.715773	0.054*	
N6	0.1512 (3)	0.49363 (17)	0.60548 (15)	0.0415 (5)	
N7	0.1432 (4)	0.7136 (2)	0.7607 (2)	0.0749 (9)	
N8	0.3283 (3)	0.57007 (18)	0.47862 (17)	0.0509 (6)	
H8A	0.292853	0.502581	0.467258	0.061*	
H8B	0.393353	0.591571	0.438508	0.061*	
N9	0.2321 (4)	0.8553 (2)	0.0099 (2)	0.0683 (8)	
N10	0.2728 (3)	0.2365 (2)	0.29056 (19)	0.0576 (7)	
C1	0.6661 (3)	0.82926 (19)	0.24815 (18)	0.0397 (6)	
C2	0.7397 (4)	0.8984 (2)	0.1980 (2)	0.0491 (7)	
H2A	0.775447	0.874291	0.142162	0.059*	
C3	0.7590 (4)	1.0028 (2)	0.2316 (2)	0.0579 (8)	
H3A	0.806985	1.049024	0.197694	0.069*	
C4	0.7085 (5)	1.0401 (2)	0.3146 (2)	0.0636 (9)	
H4A	0.722721	1.110754	0.336468	0.076*	
C5	0.6365 (4)	0.9715 (2)	0.3652 (2)	0.0536 (7)	
H5A	0.602677	0.996593	0.421230	0.064*	
C6	0.6139 (3)	0.8654 (2)	0.33319 (19)	0.0420 (6)	
C7	0.6755 (3)	0.5866 (2)	0.10982 (18)	0.0397 (6)	
C8	0.7337 (4)	0.5420 (2)	0.02367 (19)	0.0461 (6)	
C9	0.5411 (4)	0.7930 (2)	0.38984 (19)	0.0453 (6)	
C10	0.5941 (4)	0.5167 (2)	0.1609 (2)	0.0485 (7)	
C11	0.0087 (3)	0.3522 (2)	0.65460 (18)	0.0398 (6)	
C12	0.0409 (4)	0.2854 (2)	0.5782 (2)	0.0478 (7)	
H12A	0.100522	0.310240	0.534674	0.057*	
C13	−0.0153 (4)	0.1830 (2)	0.5672 (2)	0.0577 (8)	
H13A	0.005908	0.138834	0.515929	0.069*	
C14	−0.1030 (5)	0.1447 (2)	0.6314 (2)	0.0624 (9)	
H14A	−0.138944	0.074782	0.623994	0.075*	
C15	−0.1371 (4)	0.2102 (2)	0.7063 (2)	0.0528 (7)	
H15A	−0.197101	0.183987	0.749065	0.063*	
C16	−0.0837 (3)	0.3151 (2)	0.71983 (19)	0.0419 (6)	
C17	0.2032 (3)	0.5910 (2)	0.61849 (18)	0.0395 (6)	
C18	0.3021 (3)	0.6324 (2)	0.55211 (18)	0.0409 (6)	
C19	−0.1254 (4)	0.3835 (2)	0.8002 (2)	0.0465 (6)	
C20	0.1723 (4)	0.6620 (2)	0.6966 (2)	0.0484 (7)	
C21	0.0905 (5)	0.7971 (3)	−0.0140 (2)	0.0663 (9)	
H21A	0.098251	0.735057	−0.052137	0.080*	
C23	0.2309 (7)	0.9502 (4)	0.0710 (4)	0.1176 (19)	
H23A	0.126554	0.953456	0.097600	0.176*	
H23B	0.240655	1.009384	0.037917	0.176*	
H23C	0.324543	0.951311	0.118398	0.176*	
C22	0.3904 (6)	0.8209 (4)	−0.0208 (4)	0.1016 (15)	
H22A	0.371200	0.756777	−0.061050	0.152*	
H22B	0.470003	0.809503	0.030799	0.152*	
H22C	0.435242	0.873648	−0.052721	0.152*	
C24	0.2762 (4)	0.3327 (2)	0.3335 (2)	0.0605 (8)	
H24A	0.326115	0.384986	0.306639	0.073*	
C25	0.3442 (7)	0.2137 (3)	0.2058 (3)	0.0922 (13)	
H25A	0.389979	0.277101	0.189053	0.138*	
H25B	0.257282	0.184864	0.158807	0.138*	
H25C	0.432807	0.164055	0.213278	0.138*	
C26	0.2027 (6)	0.1503 (3)	0.3292 (3)	0.0849 (12)	
H26A	0.160269	0.176165	0.385159	0.127*	
H26B	0.289377	0.100539	0.341305	0.127*	
H26C	0.111800	0.117008	0.287035	0.127*	

### Atomic displacement parameters (Å^2^)

**Table:**

	*U*^11^	*U*^22^	*U*^33^	*U*^12^	*U*^13^	*U*^23^
O1	0.0856 (16)	0.0436 (11)	0.0549 (13)	−0.0106 (10)	0.0279 (11)	−0.0039 (9)
O2	0.0690 (13)	0.0373 (10)	0.0534 (12)	−0.0072 (9)	0.0216 (10)	−0.0006 (9)
O3	0.0758 (15)	0.0416 (11)	0.0537 (12)	−0.0098 (10)	0.0228 (11)	−0.0031 (9)
O4	0.0814 (16)	0.0479 (12)	0.0592 (13)	−0.0111 (11)	0.0265 (11)	0.0033 (10)
O5	0.0795 (15)	0.0445 (12)	0.0611 (13)	−0.0106 (10)	0.0271 (11)	−0.0018 (10)
O6	0.0711 (14)	0.0408 (11)	0.0515 (12)	−0.0104 (9)	0.0188 (10)	−0.0006 (9)
O7	0.0848 (19)	0.0619 (15)	0.099 (2)	−0.0123 (13)	0.0337 (16)	−0.0017 (14)
O8	0.108 (2)	0.0609 (15)	0.0751 (17)	−0.0173 (14)	0.0416 (15)	−0.0157 (13)
N1	0.0473 (13)	0.0356 (12)	0.0453 (13)	−0.0052 (9)	0.0113 (10)	0.0018 (9)
N2	0.0433 (12)	0.0364 (11)	0.0439 (12)	−0.0036 (9)	0.0074 (10)	0.0017 (9)
N3	0.086 (2)	0.0595 (17)	0.073 (2)	−0.0141 (15)	0.0230 (17)	0.0118 (15)
N4	0.087 (2)	0.0456 (14)	0.0581 (16)	−0.0191 (13)	0.0296 (14)	−0.0031 (12)
N5	0.0528 (14)	0.0381 (12)	0.0449 (13)	−0.0049 (10)	0.0123 (11)	0.0003 (10)
N6	0.0450 (12)	0.0378 (12)	0.0419 (12)	−0.0002 (9)	0.0071 (10)	0.0042 (9)
N7	0.097 (2)	0.0653 (19)	0.0621 (18)	−0.0025 (16)	0.0248 (17)	−0.0080 (15)
N8	0.0624 (16)	0.0403 (13)	0.0517 (14)	−0.0032 (11)	0.0197 (12)	−0.0004 (11)
N9	0.081 (2)	0.0551 (16)	0.0695 (19)	−0.0144 (15)	0.0131 (15)	0.0069 (14)
N10	0.0616 (16)	0.0492 (15)	0.0596 (16)	−0.0057 (12)	0.0086 (13)	−0.0047 (12)
C1	0.0392 (14)	0.0327 (13)	0.0453 (15)	0.0004 (10)	0.0019 (11)	0.0004 (11)
C2	0.0531 (17)	0.0408 (15)	0.0537 (17)	−0.0052 (12)	0.0101 (13)	0.0035 (13)
C3	0.068 (2)	0.0406 (16)	0.067 (2)	−0.0084 (14)	0.0140 (16)	0.0105 (14)
C4	0.082 (2)	0.0319 (15)	0.077 (2)	−0.0048 (15)	0.0198 (19)	−0.0015 (14)
C5	0.0617 (19)	0.0377 (15)	0.0612 (19)	−0.0010 (13)	0.0153 (15)	−0.0027 (13)
C6	0.0412 (14)	0.0347 (13)	0.0498 (16)	−0.0020 (11)	0.0063 (12)	0.0035 (11)
C7	0.0430 (14)	0.0354 (13)	0.0399 (14)	−0.0037 (11)	0.0056 (11)	0.0014 (11)
C8	0.0507 (16)	0.0415 (15)	0.0453 (15)	−0.0067 (12)	0.0077 (12)	0.0003 (12)
C9	0.0445 (15)	0.0421 (15)	0.0491 (16)	−0.0018 (12)	0.0105 (12)	−0.0010 (12)
C10	0.0520 (17)	0.0412 (15)	0.0508 (17)	−0.0084 (12)	0.0083 (13)	−0.0018 (13)
C11	0.0387 (14)	0.0357 (13)	0.0440 (15)	0.0014 (10)	0.0010 (11)	0.0053 (11)
C12	0.0528 (17)	0.0397 (15)	0.0516 (17)	0.0020 (12)	0.0085 (13)	0.0060 (12)
C13	0.070 (2)	0.0430 (16)	0.0576 (19)	0.0028 (14)	0.0082 (16)	−0.0044 (14)
C14	0.074 (2)	0.0357 (15)	0.076 (2)	−0.0067 (14)	0.0094 (18)	0.0021 (15)
C15	0.0549 (18)	0.0445 (16)	0.0602 (19)	−0.0029 (13)	0.0079 (14)	0.0108 (14)
C16	0.0411 (14)	0.0380 (14)	0.0462 (15)	0.0003 (11)	0.0016 (12)	0.0071 (11)
C17	0.0446 (15)	0.0344 (13)	0.0395 (14)	−0.0009 (11)	0.0058 (11)	0.0033 (11)
C18	0.0446 (15)	0.0363 (14)	0.0415 (14)	0.0028 (11)	0.0057 (11)	0.0027 (11)
C19	0.0475 (16)	0.0448 (16)	0.0484 (16)	−0.0010 (12)	0.0086 (12)	0.0080 (12)
C20	0.0548 (17)	0.0411 (15)	0.0496 (17)	−0.0029 (12)	0.0114 (13)	0.0021 (13)
C21	0.086 (3)	0.0492 (18)	0.065 (2)	−0.0128 (17)	0.0207 (19)	0.0022 (15)
C23	0.133 (4)	0.073 (3)	0.137 (5)	−0.029 (3)	0.019 (4)	−0.029 (3)
C22	0.079 (3)	0.109 (4)	0.116 (4)	−0.011 (3)	0.015 (3)	0.011 (3)
C24	0.066 (2)	0.0469 (17)	0.068 (2)	−0.0111 (15)	0.0156 (17)	−0.0022 (15)
C25	0.127 (4)	0.075 (3)	0.074 (3)	0.001 (2)	0.031 (3)	−0.013 (2)
C26	0.109 (3)	0.051 (2)	0.094 (3)	−0.014 (2)	0.018 (2)	−0.0004 (19)

### Geometric parameters (Å, º)

**Table:**

O1—C9	1.317 (3)	C3—C4	1.379 (5)
O1—H1A	0.8502	C3—H3A	0.9300
O2—C9	1.228 (3)	C4—C5	1.385 (4)
O3—C8	1.247 (3)	C4—H4A	0.9300
O4—C19	1.311 (3)	C5—C6	1.394 (4)
O4—H4B	0.8501	C5—H5A	0.9300
O5—C19	1.230 (3)	C6—C9	1.483 (4)
O6—C18	1.249 (3)	C7—C10	1.438 (4)
O7—C21	1.219 (4)	C7—C8	1.471 (4)
O8—C24	1.219 (4)	C11—C12	1.394 (4)
N1—N2	1.325 (3)	C11—C16	1.407 (4)
N1—C1	1.393 (3)	C12—C13	1.371 (4)
N1—H1N	0.8999	C12—H12A	0.9300
N2—C7	1.299 (3)	C13—C14	1.380 (5)
N3—C10	1.138 (4)	C13—H13A	0.9300
N4—C8	1.313 (4)	C14—C15	1.372 (4)
N4—H4C	0.8999	C14—H14A	0.9300
N4—H4D	0.9000	C15—C16	1.395 (4)
N5—N6	1.325 (3)	C15—H15A	0.9300
N5—C11	1.398 (3)	C16—C19	1.473 (4)
N5—H5N	0.9000	C17—C20	1.439 (4)
N6—C17	1.300 (3)	C17—C18	1.476 (4)
N7—C20	1.145 (4)	C21—H21A	0.9300
N8—C18	1.318 (3)	C23—H23A	0.9600
N8—H8A	0.9000	C23—H23B	0.9600
N8—H8B	0.9000	C23—H23C	0.9600
N9—C21	1.333 (5)	C22—H22A	0.9600
N9—C23	1.435 (5)	C22—H22B	0.9600
N9—C22	1.446 (5)	C22—H22C	0.9600
N10—C24	1.323 (4)	C24—H24A	0.9300
N10—C26	1.442 (5)	C25—H25A	0.9600
N10—C25	1.445 (5)	C25—H25B	0.9600
C1—C2	1.392 (4)	C25—H25C	0.9600
C1—C6	1.408 (4)	C26—H26A	0.9600
C2—C3	1.377 (4)	C26—H26B	0.9600
C2—H2A	0.9300	C26—H26C	0.9600
			
C9—O1—H1A	115.0	C13—C12—H12A	120.0
C19—O4—H4B	111.9	C11—C12—H12A	120.0
N2—N1—C1	120.3 (2)	C12—C13—C14	120.7 (3)
N2—N1—H1N	122.1	C12—C13—H13A	119.6
C1—N1—H1N	117.4	C14—C13—H13A	119.6
C7—N2—N1	118.8 (2)	C15—C14—C13	119.7 (3)
C8—N4—H4C	126.8	C15—C14—H14A	120.1
C8—N4—H4D	120.3	C13—C14—H14A	120.1
H4C—N4—H4D	112.9	C14—C15—C16	121.5 (3)
N6—N5—C11	120.4 (2)	C14—C15—H15A	119.2
N6—N5—H5N	121.4	C16—C15—H15A	119.2
C11—N5—H5N	118.1	C15—C16—C11	118.0 (3)
C17—N6—N5	119.1 (2)	C15—C16—C19	119.9 (3)
C18—N8—H8A	124.0	C11—C16—C19	122.1 (2)
C18—N8—H8B	120.7	N6—C17—C20	123.2 (2)
H8A—N8—H8B	115.1	N6—C17—C18	119.7 (2)
C21—N9—C23	120.1 (4)	C20—C17—C18	117.1 (2)
C21—N9—C22	120.7 (3)	O6—C18—N8	124.0 (3)
C23—N9—C22	119.1 (4)	O6—C18—C17	117.6 (2)
C24—N10—C26	120.5 (3)	N8—C18—C17	118.4 (2)
C24—N10—C25	121.4 (3)	O5—C19—O4	121.9 (3)
C26—N10—C25	118.1 (3)	O5—C19—C16	123.8 (3)
C2—C1—N1	120.9 (2)	O4—C19—C16	114.3 (2)
C2—C1—C6	120.4 (2)	N7—C20—C17	175.9 (3)
N1—C1—C6	118.7 (2)	O7—C21—N9	125.8 (3)
C3—C2—C1	119.4 (3)	O7—C21—H21A	117.1
C3—C2—H2A	120.3	N9—C21—H21A	117.1
C1—C2—H2A	120.3	N9—C23—H23A	109.5
C2—C3—C4	121.3 (3)	N9—C23—H23B	109.5
C2—C3—H3A	119.4	H23A—C23—H23B	109.5
C4—C3—H3A	119.4	N9—C23—H23C	109.5
C3—C4—C5	119.6 (3)	H23A—C23—H23C	109.5
C3—C4—H4A	120.2	H23B—C23—H23C	109.5
C5—C4—H4A	120.2	N9—C22—H22A	109.5
C4—C5—C6	120.9 (3)	N9—C22—H22B	109.5
C4—C5—H5A	119.6	H22A—C22—H22B	109.5
C6—C5—H5A	119.6	N9—C22—H22C	109.5
C5—C6—C1	118.5 (3)	H22A—C22—H22C	109.5
C5—C6—C9	119.9 (3)	H22B—C22—H22C	109.5
C1—C6—C9	121.6 (2)	O8—C24—N10	125.8 (3)
N2—C7—C10	121.8 (2)	O8—C24—H24A	117.1
N2—C7—C8	120.5 (2)	N10—C24—H24A	117.1
C10—C7—C8	117.7 (2)	N10—C25—H25A	109.5
O3—C8—N4	123.7 (3)	N10—C25—H25B	109.5
O3—C8—C7	118.1 (2)	H25A—C25—H25B	109.5
N4—C8—C7	118.3 (2)	N10—C25—H25C	109.5
O2—C9—O1	122.1 (3)	H25A—C25—H25C	109.5
O2—C9—C6	123.8 (2)	H25B—C25—H25C	109.5
O1—C9—C6	114.1 (2)	N10—C26—H26A	109.5
N3—C10—C7	173.2 (3)	N10—C26—H26B	109.5
C12—C11—N5	120.5 (2)	H26A—C26—H26B	109.5
C12—C11—C16	120.1 (2)	N10—C26—H26C	109.5
N5—C11—C16	119.4 (2)	H26A—C26—H26C	109.5
C13—C12—C11	120.0 (3)	H26B—C26—H26C	109.5
			
C1—N1—N2—C7	179.7 (2)	N6—N5—C11—C16	−179.2 (2)
C11—N5—N6—C17	−179.8 (2)	N5—C11—C12—C13	179.8 (3)
N2—N1—C1—C2	2.6 (4)	C16—C11—C12—C13	−0.9 (4)
N2—N1—C1—C6	−177.1 (2)	C11—C12—C13—C14	−0.4 (5)
N1—C1—C2—C3	179.5 (3)	C12—C13—C14—C15	1.2 (5)
C6—C1—C2—C3	−0.9 (4)	C13—C14—C15—C16	−0.6 (5)
C1—C2—C3—C4	0.7 (5)	C14—C15—C16—C11	−0.7 (4)
C2—C3—C4—C5	−0.2 (5)	C14—C15—C16—C19	178.9 (3)
C3—C4—C5—C6	−0.2 (5)	C12—C11—C16—C15	1.5 (4)
C4—C5—C6—C1	0.0 (5)	N5—C11—C16—C15	−179.3 (2)
C4—C5—C6—C9	178.2 (3)	C12—C11—C16—C19	−178.1 (3)
C2—C1—C6—C5	0.5 (4)	N5—C11—C16—C19	1.1 (4)
N1—C1—C6—C5	−179.8 (3)	N5—N6—C17—C20	−0.1 (4)
C2—C1—C6—C9	−177.7 (3)	N5—N6—C17—C18	178.5 (2)
N1—C1—C6—C9	2.0 (4)	N6—C17—C18—O6	−177.4 (3)
N1—N2—C7—C10	−0.1 (4)	C20—C17—C18—O6	1.3 (4)
N1—N2—C7—C8	179.9 (2)	N6—C17—C18—N8	3.1 (4)
N2—C7—C8—O3	−178.4 (3)	C20—C17—C18—N8	−178.1 (3)
C10—C7—C8—O3	1.6 (4)	C15—C16—C19—O5	178.7 (3)
N2—C7—C8—N4	1.6 (4)	C11—C16—C19—O5	−1.7 (4)
C10—C7—C8—N4	−178.3 (3)	C15—C16—C19—O4	−2.9 (4)
C5—C6—C9—O2	178.2 (3)	C11—C16—C19—O4	176.7 (3)
C1—C6—C9—O2	−3.6 (4)	C23—N9—C21—O7	2.1 (6)
C5—C6—C9—O1	−1.8 (4)	C22—N9—C21—O7	178.5 (4)
C1—C6—C9—O1	176.4 (3)	C26—N10—C24—O8	−2.1 (6)
N6—N5—C11—C12	0.0 (4)	C25—N10—C24—O8	−179.8 (4)

### Hydrogen-bond geometry (Å, º)

**Table:**

*D*—H···*A*	*D*—H	H···*A*	*D*···*A*	*D*—H···*A*
O1—H1*A*···O6	0.85	1.73	2.580 (3)	175
O4—H4*B*···O3^i^	0.85	1.75	2.590 (3)	171
N1—H1*N*···O2	0.90	1.89	2.617 (3)	136
N4—H4*C*···O7^ii^	0.90	2.04	2.915 (3)	162
N4—H4*D*···O5^iii^	0.90	2.09	2.952 (3)	161
N5—H5*N*···O5	0.90	1.93	2.640 (3)	134
N8—H8*A*···O8	0.90	2.01	2.889 (3)	164
N8—H8*B*···O2	0.90	2.14	2.990 (3)	158
C2—H2*A*···O7^ii^	0.93	2.60	3.505 (4)	165
C4—H4*A*···O6^iv^	0.93	2.52	3.372 (3)	153
C12—H12*A*···O8	0.93	2.39	3.311 (4)	171
C24—H24*A*···N3	0.93	2.62	3.498 (4)	158

Symmetry codes: (i) *x*−1, *y*, *z*+1; (ii) *x*+1, *y*, *z*; (iii) *x*+1, *y*, *z*−1; (iv) −*x*+1, −*y*+2, −*z*+1.
